# 2-[(1,3-Benzodioxol-5-yl­methyl­idene)amino]-4,5,6,7-tetra­hydro-1-benzothio­phene-3-carbonitrile

**DOI:** 10.1107/S1600536811029564

**Published:** 2011-07-30

**Authors:** Abdullah M. Asiri, Salman A. Khan, M. Nawaz Tahir

**Affiliations:** a The Center of Excellence for Advanced Materials Research, King Abdulaziz University, Jeddah 21589, PO Box 80203, Saudi Arabia; b Department of Chemistry, Faculty of Science, King Abduaziz University, Jeddah 21589, PO Box 80203, Saudi Arabia; c University of Sargodha, Department of Physics, Sargodha, Pakistan

## Abstract

The title compound, C_17_H_14_N_2_O_2_S, crystallizes with two roughly planar mol­ecules in the asymmetric unit, in which the dihedral angles between the 1,3-benzodioxole-5-carbaldehyde moiety and the heterocyclic five-membered ring are 3.76 (5) and 5.33 (12)°. In each mol­ecule, a short C—H⋯S contact generates an *S*(5) ring. In the crystal, pairs of mol­ecules are linked by a weak C—H⋯N inter­action, forming dimers.

## Related literature

For a related structure, see: Elerman & Elmali, (1998 ▶). For graph-set notation, see: Bernstein *et al.* (1995 ▶).
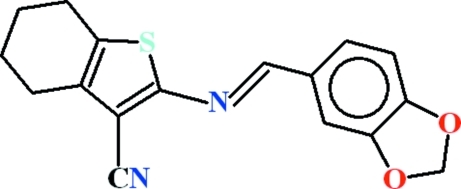


## Experimental

### 

#### Crystal data

C_17_H_14_N_2_O_2_S
*M* *_r_* = 310.36
Triclinic, 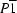

*a* = 10.9450 (3) Å
*b* = 10.9895 (3) Å
*c* = 13.5749 (3) Å
α = 99.409 (1)°
β = 109.707 (1)°
γ = 92.854 (1)°
*V* = 1506.77 (7) Å^3^
*Z* = 4
Mo *K*α radiation
μ = 0.22 mm^−1^
*T* = 296 K
0.32 × 0.23 × 0.20 mm

#### Data collection

Bruker Kappa APEXII CCD diffractometer
Absorption correction: multi-scan (*SADABS*; Bruker, 2005 ▶) *T*_min_ = 0.947, *T*_max_ = 0.962
21604 measured reflections
5331 independent reflections
3812 reflections with *I* > 2σ(*I*)
*R*_int_ = 0.030

#### Refinement

*R*[*F* ^2^ > 2σ(*F* ^2^)] = 0.042
*wR*(*F* ^2^) = 0.119
*S* = 1.01
5331 reflections
397 parameters
H-atom parameters constrained
Δρ_max_ = 0.47 e Å^−3^
Δρ_min_ = −0.19 e Å^−3^

### 

Data collection: *APEX2* (Bruker, 2009 ▶); cell refinement: *SAINT* (Bruker, 2009 ▶); data reduction: *SAINT*; program(s) used to solve structure: *SHELXS97* (Sheldrick, 2008 ▶); program(s) used to refine structure: *SHELXL97* (Sheldrick, 2008 ▶); molecular graphics: *ORTEP-3 for Windows* (Farrugia, 1997 ▶) and *PLATON* (Spek, 2009 ▶); software used to prepare material for publication: *WinGX* (Farrugia, 1999 ▶) and *PLATON*.

## Supplementary Material

Crystal structure: contains datablock(s) global, I. DOI: 10.1107/S1600536811029564/hb6325sup1.cif

Structure factors: contains datablock(s) I. DOI: 10.1107/S1600536811029564/hb6325Isup2.hkl

Supplementary material file. DOI: 10.1107/S1600536811029564/hb6325Isup3.cml

Additional supplementary materials:  crystallographic information; 3D view; checkCIF report

## Figures and Tables

**Table 1:** Hydrogen-bond geometry (Å, °)

*D*—H⋯*A*	*D*—H	H⋯*A*	*D*⋯*A*	*D*—H⋯*A*
C8—H8⋯S1	0.93	2.65	3.081 (2)	109
C25—H25⋯S2	0.93	2.61	3.060 (2)	110
C7—H7*A*⋯N4^i^	0.97	2.62	3.190 (3)	118

Symmetry code: (i) 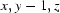.

**Fig. 1.**
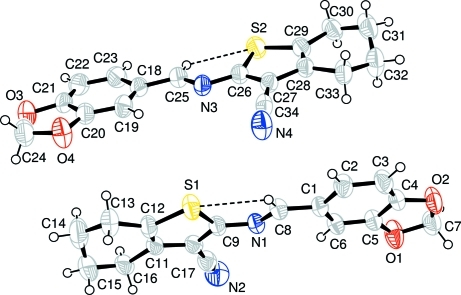
View of the title compound with displacement ellipsoids drawn at the 50% probability level. The dotted lines represent the C—H···S short contacts.

**Fig. 2.**
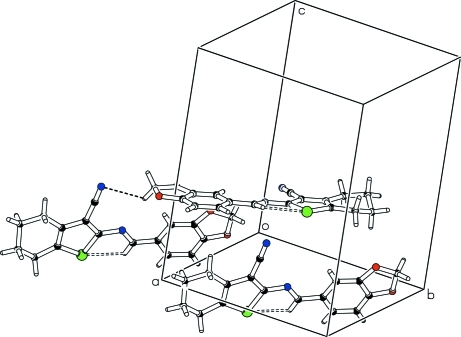
The partial packing of (I0, which shows that molecules are linked into pairs.

## supplementary crystallographic information

### Comment

The crystal structures of 2-salicylideneamino-4,5,6,7-tetrahydrobenzo(*b*) thiophene-3-carbonitrile (Elerman & Elmali, 1998) has been published which is related to the title compound (I, Fig. 1).

The title compound consist of two molecules having different configuration. In one molecule, the ring system of 1,3-benzodioxole-5-carbaldehyde moiety A (C1—C7/O1/O2) and five membered ring B (C9—C12/S1) of 2-amino-4,5,6,7- tetrahydro-1-benzothiophene-3-carbonitrile group are planar with r. m. s. deviations of 0.010 and 0.007 Å, respectively. The dihedral angle between A/B is 3.76 (5)°. In the second molecule, the ring system of 1,3-benzodioxole-5-carbaldehyde moiety C (C18—C24/O3/O4) and five membered ring D (C26—C29/S2) of 2-amino-4,5,6,7-tetrahydro-1-benzothiophene-3- carbonitrile group are also almost planar with r. m. s. deviation of 0.003 and 0.003 Å, respectively. The dihedral angle between C/D is 5.33 (12)°. There exist intra-molecular H-bonding of C—H···S type completing S(5) ring (Table 1, Fig. 1) motifs (Bernstein *et al.*, 1995) in each molecule. The inter-molecular H-bondings of C—H···N type links the molecules in pair.

### Experimental

A mixture of 1,3-benzodioxole-5-carbaldehyde (0.50 g, 3.3 mmol) and 2-amino-4,5,6,7-tetrahydro-1-benzothiophene-3-carbonitrile (0.58 g, 3.3 mmol) in ethanol (15 ml) was heated for 3 h. The progress of the reaction was monitored by TLC. The solid that separated from the cooled mixture was collected and recrystallized from a methanol-chloroform mixture (9:1) to give yellow prisms of the title compound (I).

Yield: 80%; m.p. 452–453 K.

### Refinement

The H-atoms were positioned geometrically (C–H = 0.93–0.97 Å) and refined as riding with *U*_iso_(H) = x*U*_eq_(C), where x = 1.2 for all H-atoms.

### Crystal data

**Table:**

C_17_H_14_N_2_O_2_S	*Z* = 4
*M**_r_* = 310.36	*F*(000) = 648
Triclinic, *P*1	*D*_x_ = 1.368 Mg m^−^^3^
Hall symbol: -P 1	Mo *K*α radiation, λ = 0.71073 Å
*a* = 10.9450 (3) Å	Cell parameters from 3812 reflections
*b* = 10.9895 (3) Å	θ = 3.0–25.3°
*c* = 13.5749 (3) Å	µ = 0.22 mm^−^^1^
α = 99.409 (1)°	*T* = 296 K
β = 109.707 (1)°	Prism, yellow
γ = 92.854 (1)°	0.32 × 0.23 × 0.20 mm
*V* = 1506.77 (7) Å^3^	

### Data collection

**Table:**

Bruker Kappa APEXII CCD diffractometer	5331 independent reflections
Radiation source: fine-focus sealed tube	3812 reflections with *I* > 2σ(*I*)
graphite	*R*_int_ = 0.030
Detector resolution: 8.20 pixels mm^-1^	θ_max_ = 25.1°, θ_min_ = 3.0°
ω scans	*h* = −13→13
Absorption correction: multi-scan (*SADABS*; Bruker, 2005)	*k* = −13→13
*T*_min_ = 0.947, *T*_max_ = 0.962	*l* = −16→16
21604 measured reflections	

### Refinement

**Table:**

Refinement on *F*^2^	Primary atom site location: structure-invariant direct methods
Least-squares matrix: full	Secondary atom site location: difference Fourier map
*R*[*F*^2^ > 2σ(*F*^2^)] = 0.042	Hydrogen site location: inferred from neighbouring sites
*wR*(*F*^2^) = 0.119	H-atom parameters constrained
*S* = 1.01	*w* = 1/[σ^2^(*F*_o_^2^) + (0.0574*P*)^2^ + 0.3989*P*] where *P* = (*F*_o_^2^ + 2*F*_c_^2^)/3
5331 reflections	(Δ/σ)_max_ < 0.001
397 parameters	Δρ_max_ = 0.47 e Å^−^^3^
0 restraints	Δρ_min_ = −0.19 e Å^−^^3^

### Special details

**Table:**

Geometry. Bond distances, angles *etc*. have been calculated using the rounded fractional coordinates. All su's are estimated from the variances of the (full) variance-covariance matrix. The cell e.s.d.'s are taken into account in the estimation of distances, angles and torsion angles
Refinement. Refinement of *F*^2^ against ALL reflections. The weighted *R*-factor *wR* and goodness of fit *S* are based on *F*^2^, conventional *R*-factors *R* are based on *F*, with *F* set to zero for negative *F*^2^. The threshold expression of *F*^2^ > σ(*F*^2^) is used only for calculating *R*-factors(gt) *etc*. and is not relevant to the choice of reflections for refinement. *R*-factors based on *F*^2^ are statistically about twice as large as those based on *F*, and *R*- factors based on ALL data will be even larger.

### Fractional atomic coordinates and isotropic or equivalent isotropic displacement parameters (Å^2^)

**Table:**

	*x*	*y*	*z*	*U*_iso_*/*U*_eq_	
S1	0.69931 (6)	0.61842 (6)	0.38319 (5)	0.0608 (3)	
O1	0.83134 (17)	−0.08541 (15)	0.34616 (16)	0.0760 (7)	
O2	1.04492 (17)	−0.06592 (16)	0.35358 (15)	0.0732 (7)	
N1	0.68413 (17)	0.36120 (16)	0.35774 (13)	0.0469 (6)	
N2	0.3467 (2)	0.2739 (2)	0.32998 (19)	0.0791 (9)	
C1	0.87133 (19)	0.25306 (19)	0.37004 (16)	0.0454 (7)	
C2	1.0002 (2)	0.2632 (2)	0.37598 (19)	0.0574 (8)	
C3	1.0676 (2)	0.1591 (2)	0.3701 (2)	0.0636 (10)	
C4	1.0009 (2)	0.0478 (2)	0.35961 (18)	0.0523 (8)	
C5	0.8728 (2)	0.0370 (2)	0.35467 (17)	0.0495 (8)	
C6	0.8052 (2)	0.13625 (19)	0.35878 (17)	0.0484 (7)	
C7	0.9391 (3)	−0.1520 (2)	0.3461 (2)	0.0698 (10)	
C8	0.8065 (2)	0.3644 (2)	0.37473 (17)	0.0489 (8)	
C9	0.6260 (2)	0.4690 (2)	0.36425 (16)	0.0463 (7)	
C10	0.4990 (2)	0.4719 (2)	0.35984 (16)	0.0465 (7)	
C11	0.4612 (2)	0.5942 (2)	0.37466 (16)	0.0490 (8)	
C12	0.5596 (2)	0.6817 (2)	0.38799 (18)	0.0552 (8)	
C13	0.5532 (3)	0.8189 (2)	0.4071 (2)	0.0745 (10)	
C14	0.4109 (3)	0.8429 (3)	0.3783 (3)	0.0969 (14)	
C15	0.3357 (3)	0.7633 (3)	0.4195 (3)	0.0927 (12)	
C16	0.3315 (2)	0.6258 (2)	0.37836 (19)	0.0625 (9)	
C17	0.4154 (2)	0.3617 (2)	0.34364 (18)	0.0553 (9)	
S2	0.74553 (6)	0.38875 (6)	−0.09354 (5)	0.0621 (2)	
O3	0.33292 (19)	0.99165 (18)	0.05740 (17)	0.0814 (8)	
O4	0.51968 (18)	0.97170 (16)	0.19428 (15)	0.0798 (7)	
N3	0.71457 (18)	0.58138 (16)	0.05320 (15)	0.0509 (7)	
N4	0.9964 (2)	0.5672 (2)	0.29555 (19)	0.0721 (8)	
C18	0.5351 (2)	0.7022 (2)	0.00214 (17)	0.0494 (8)	
C19	0.5760 (2)	0.7860 (2)	0.09919 (18)	0.0537 (8)	
C20	0.5008 (2)	0.8788 (2)	0.10818 (19)	0.0538 (8)	
C21	0.3895 (2)	0.8915 (2)	0.0267 (2)	0.0571 (9)	
C22	0.3474 (2)	0.8116 (3)	−0.0684 (2)	0.0675 (10)	
C23	0.4220 (2)	0.7156 (2)	−0.07933 (19)	0.0620 (9)	
C24	0.4140 (3)	1.0438 (3)	0.1634 (3)	0.0790 (11)	
C25	0.6096 (2)	0.6012 (2)	−0.01630 (19)	0.0542 (8)	
C26	0.7833 (2)	0.48621 (19)	0.02932 (18)	0.0495 (8)	
C27	0.8952 (2)	0.45635 (19)	0.10042 (17)	0.0478 (8)	
C28	0.9499 (2)	0.35489 (19)	0.05702 (19)	0.0502 (8)	
C29	0.8797 (2)	0.3105 (2)	−0.0468 (2)	0.0550 (8)	
C30	0.9103 (3)	0.2036 (2)	−0.1155 (2)	0.0675 (10)	
C31	1.0462 (3)	0.1722 (3)	−0.0606 (3)	0.0790 (11)	
C32	1.0763 (3)	0.1751 (3)	0.0558 (3)	0.0878 (11)	
C33	1.0699 (3)	0.3015 (2)	0.1166 (2)	0.0649 (9)	
C34	0.9495 (2)	0.5197 (2)	0.2080 (2)	0.0535 (9)	
H2	1.04310	0.34134	0.38414	0.0689*	
H3	1.15386	0.16599	0.37317	0.0763*	
H6	0.71841	0.12736	0.35432	0.0581*	
H7A	0.91573	−0.21367	0.28101	0.0838*	
H7B	0.96394	−0.19426	0.40615	0.0838*	
H8	0.85578	0.44114	0.39070	0.0587*	
H13A	0.59244	0.85699	0.36364	0.0894*	
H13B	0.60114	0.85481	0.48136	0.0894*	
H14A	0.40709	0.92904	0.40645	0.1157*	
H14B	0.37063	0.82949	0.30131	0.1157*	
H15A	0.24704	0.78525	0.40004	0.1114*	
H15B	0.37364	0.77969	0.49667	0.1114*	
H16A	0.30649	0.57872	0.42448	0.0750*	
H16B	0.26608	0.60265	0.30742	0.0750*	
H19	0.65114	0.77860	0.15506	0.0645*	
H22	0.27214	0.82079	−0.12347	0.0811*	
H23	0.39555	0.65842	−0.14321	0.0744*	
H24A	0.36445	1.04391	0.21071	0.0947*	
H24B	0.44678	1.12889	0.16768	0.0947*	
H25	0.57911	0.54799	−0.08239	0.0650*	
H30A	0.90390	0.22530	−0.18334	0.0810*	
H30B	0.84742	0.13191	−0.12909	0.0810*	
H31A	1.05551	0.09013	−0.09447	0.0950*	
H31B	1.10907	0.23096	−0.06945	0.0950*	
H32A	1.16308	0.15071	0.08613	0.1056*	
H32B	1.01468	0.11488	0.06452	0.1056*	
H33A	1.06867	0.29412	0.18647	0.0778*	
H33B	1.14680	0.35662	0.12626	0.0778*	

### Atomic displacement parameters (Å^2^)

**Table:**

	*U*^11^	*U*^22^	*U*^33^	*U*^12^	*U*^13^	*U*^23^
S1	0.0483 (4)	0.0490 (4)	0.0878 (5)	0.0129 (3)	0.0275 (3)	0.0109 (3)
O1	0.0641 (11)	0.0413 (9)	0.1252 (16)	0.0107 (8)	0.0392 (11)	0.0086 (9)
O2	0.0650 (11)	0.0577 (11)	0.1085 (14)	0.0299 (9)	0.0416 (10)	0.0167 (10)
N1	0.0427 (11)	0.0475 (11)	0.0511 (10)	0.0126 (8)	0.0173 (8)	0.0073 (8)
N2	0.0574 (14)	0.0806 (17)	0.0976 (18)	−0.0021 (13)	0.0271 (13)	0.0153 (14)
C1	0.0384 (12)	0.0472 (13)	0.0500 (12)	0.0104 (10)	0.0146 (10)	0.0078 (10)
C2	0.0417 (13)	0.0537 (14)	0.0791 (16)	0.0074 (11)	0.0216 (12)	0.0175 (12)
C3	0.0435 (13)	0.0665 (17)	0.0918 (19)	0.0174 (12)	0.0326 (13)	0.0227 (14)
C4	0.0496 (14)	0.0513 (14)	0.0616 (14)	0.0190 (11)	0.0245 (11)	0.0114 (10)
C5	0.0467 (13)	0.0445 (13)	0.0577 (13)	0.0080 (10)	0.0201 (10)	0.0061 (10)
C6	0.0364 (12)	0.0487 (13)	0.0591 (13)	0.0079 (10)	0.0177 (10)	0.0042 (10)
C7	0.0760 (18)	0.0537 (16)	0.0867 (19)	0.0237 (14)	0.0357 (15)	0.0122 (13)
C8	0.0439 (13)	0.0453 (13)	0.0576 (13)	0.0080 (10)	0.0184 (10)	0.0078 (10)
C9	0.0441 (12)	0.0480 (13)	0.0473 (12)	0.0133 (10)	0.0163 (10)	0.0076 (9)
C10	0.0400 (12)	0.0569 (14)	0.0424 (12)	0.0113 (10)	0.0133 (9)	0.0098 (10)
C11	0.0473 (13)	0.0600 (14)	0.0428 (12)	0.0205 (11)	0.0161 (10)	0.0135 (10)
C12	0.0538 (14)	0.0542 (14)	0.0572 (14)	0.0231 (12)	0.0177 (11)	0.0087 (11)
C13	0.0763 (18)	0.0552 (16)	0.091 (2)	0.0270 (14)	0.0269 (15)	0.0110 (14)
C14	0.098 (2)	0.078 (2)	0.124 (3)	0.0478 (19)	0.044 (2)	0.0229 (19)
C15	0.090 (2)	0.107 (2)	0.109 (2)	0.062 (2)	0.056 (2)	0.037 (2)
C16	0.0521 (14)	0.0878 (19)	0.0579 (14)	0.0340 (13)	0.0246 (12)	0.0233 (13)
C17	0.0413 (13)	0.0661 (17)	0.0595 (14)	0.0121 (12)	0.0177 (11)	0.0127 (12)
S2	0.0629 (4)	0.0661 (4)	0.0598 (4)	0.0201 (3)	0.0237 (3)	0.0105 (3)
O3	0.0814 (13)	0.0794 (13)	0.1002 (15)	0.0480 (11)	0.0408 (12)	0.0329 (11)
O4	0.0764 (12)	0.0626 (11)	0.0894 (13)	0.0246 (9)	0.0205 (10)	−0.0020 (10)
N3	0.0487 (11)	0.0472 (11)	0.0616 (12)	0.0142 (9)	0.0218 (9)	0.0156 (9)
N4	0.0724 (15)	0.0575 (13)	0.0759 (15)	0.0195 (11)	0.0162 (12)	0.0015 (12)
C18	0.0439 (12)	0.0517 (13)	0.0568 (14)	0.0121 (10)	0.0193 (11)	0.0169 (11)
C19	0.0432 (13)	0.0528 (14)	0.0612 (14)	0.0118 (10)	0.0107 (11)	0.0146 (11)
C20	0.0524 (14)	0.0480 (14)	0.0651 (15)	0.0112 (11)	0.0238 (12)	0.0138 (11)
C21	0.0526 (14)	0.0602 (15)	0.0724 (16)	0.0247 (12)	0.0294 (13)	0.0298 (13)
C22	0.0542 (15)	0.091 (2)	0.0639 (16)	0.0314 (14)	0.0168 (13)	0.0347 (15)
C23	0.0585 (15)	0.0761 (17)	0.0515 (14)	0.0202 (13)	0.0156 (12)	0.0165 (12)
C24	0.082 (2)	0.0575 (16)	0.111 (2)	0.0248 (15)	0.0473 (19)	0.0190 (16)
C25	0.0538 (14)	0.0526 (14)	0.0598 (14)	0.0127 (11)	0.0227 (12)	0.0131 (11)
C26	0.0488 (13)	0.0446 (12)	0.0611 (14)	0.0106 (10)	0.0245 (11)	0.0142 (10)
C27	0.0478 (13)	0.0395 (12)	0.0604 (14)	0.0072 (10)	0.0231 (11)	0.0115 (10)
C28	0.0494 (13)	0.0399 (12)	0.0689 (15)	0.0096 (10)	0.0285 (12)	0.0136 (10)
C29	0.0534 (14)	0.0501 (14)	0.0708 (16)	0.0109 (11)	0.0322 (12)	0.0135 (12)
C30	0.0737 (18)	0.0571 (16)	0.0816 (18)	0.0139 (13)	0.0433 (15)	0.0038 (13)
C31	0.079 (2)	0.0586 (17)	0.108 (2)	0.0217 (14)	0.0472 (17)	0.0045 (15)
C32	0.092 (2)	0.0670 (19)	0.108 (2)	0.0414 (16)	0.0351 (19)	0.0173 (16)
C33	0.0605 (16)	0.0533 (15)	0.0854 (18)	0.0226 (12)	0.0276 (14)	0.0168 (13)
C34	0.0497 (14)	0.0388 (13)	0.0737 (18)	0.0139 (10)	0.0222 (12)	0.0117 (12)

### Geometric parameters (Å, °)

**Table:**

S1—C9	1.731 (2)	C8—H8	0.9300
S1—C12	1.728 (2)	C13—H13B	0.9700
S2—C29	1.725 (2)	C13—H13A	0.9700
S2—C26	1.734 (2)	C14—H14B	0.9700
O1—C7	1.419 (4)	C14—H14A	0.9700
O1—C5	1.372 (3)	C15—H15A	0.9700
O2—C4	1.362 (3)	C15—H15B	0.9700
O2—C7	1.424 (4)	C16—H16B	0.9700
O3—C21	1.364 (3)	C16—H16A	0.9700
O3—C24	1.416 (4)	C18—C19	1.396 (3)
O4—C24	1.419 (4)	C18—C23	1.388 (3)
O4—C20	1.370 (3)	C18—C25	1.448 (3)
N1—C9	1.377 (3)	C19—C20	1.358 (3)
N1—C8	1.278 (3)	C20—C21	1.373 (3)
N2—C17	1.143 (3)	C21—C22	1.356 (4)
N3—C26	1.377 (3)	C22—C23	1.385 (4)
N3—C25	1.272 (3)	C26—C27	1.373 (3)
N4—C34	1.144 (3)	C27—C28	1.428 (3)
C1—C2	1.383 (3)	C27—C34	1.418 (3)
C1—C6	1.401 (3)	C28—C29	1.350 (3)
C1—C8	1.448 (3)	C28—C33	1.496 (4)
C2—C3	1.398 (3)	C29—C30	1.501 (4)
C3—C4	1.354 (3)	C30—C31	1.508 (5)
C4—C5	1.379 (3)	C31—C32	1.496 (5)
C5—C6	1.352 (3)	C32—C33	1.513 (4)
C9—C10	1.373 (3)	C19—H19	0.9300
C10—C11	1.429 (3)	C22—H22	0.9300
C10—C17	1.421 (3)	C23—H23	0.9300
C11—C16	1.493 (3)	C24—H24A	0.9700
C11—C12	1.351 (3)	C24—H24B	0.9700
C12—C13	1.497 (3)	C25—H25	0.9300
C13—C14	1.521 (5)	C30—H30A	0.9700
C14—C15	1.468 (5)	C30—H30B	0.9700
C15—C16	1.516 (4)	C31—H31A	0.9700
C2—H2	0.9300	C31—H31B	0.9700
C3—H3	0.9300	C32—H32A	0.9700
C6—H6	0.9300	C32—H32B	0.9700
C7—H7B	0.9700	C33—H33A	0.9700
C7—H7A	0.9700	C33—H33B	0.9700
			
C9—S1—C12	91.86 (11)	C15—C16—H16A	109.00
C26—S2—C29	92.06 (12)	C15—C16—H16B	109.00
C5—O1—C7	106.22 (19)	C11—C16—H16A	109.00
C4—O2—C7	106.0 (2)	C11—C16—H16B	109.00
C21—O3—C24	105.9 (2)	C19—C18—C23	120.1 (2)
C20—O4—C24	106.0 (2)	C19—C18—C25	121.2 (2)
C8—N1—C9	120.91 (19)	C23—C18—C25	118.7 (2)
C25—N3—C26	120.5 (2)	C18—C19—C20	116.8 (2)
C2—C1—C6	120.0 (2)	O4—C20—C19	128.0 (2)
C6—C1—C8	121.0 (2)	O4—C20—C21	109.5 (2)
C2—C1—C8	119.0 (2)	C19—C20—C21	122.5 (2)
C1—C2—C3	121.8 (2)	O3—C21—C20	110.2 (2)
C2—C3—C4	116.6 (2)	O3—C21—C22	127.8 (2)
C3—C4—C5	122.0 (2)	C20—C21—C22	122.0 (2)
O2—C4—C5	110.2 (2)	C21—C22—C23	116.6 (2)
O2—C4—C3	127.8 (2)	C18—C23—C22	122.0 (2)
O1—C5—C6	128.3 (2)	O3—C24—O4	108.5 (3)
C4—C5—C6	122.4 (2)	N3—C25—C18	123.4 (2)
O1—C5—C4	109.3 (2)	S2—C26—N3	126.02 (17)
C1—C6—C5	117.2 (2)	S2—C26—C27	109.71 (17)
O1—C7—O2	108.25 (18)	N3—C26—C27	124.3 (2)
N1—C8—C1	122.5 (2)	C26—C27—C28	114.1 (2)
S1—C9—C10	110.04 (17)	C26—C27—C34	122.6 (2)
N1—C9—C10	123.4 (2)	C28—C27—C34	123.3 (2)
S1—C9—N1	126.57 (17)	C27—C28—C29	111.8 (2)
C9—C10—C11	114.0 (2)	C27—C28—C33	125.6 (2)
C9—C10—C17	121.9 (2)	C29—C28—C33	122.7 (2)
C11—C10—C17	124.1 (2)	S2—C29—C28	112.36 (18)
C12—C11—C16	122.6 (2)	S2—C29—C30	122.51 (19)
C10—C11—C12	111.6 (2)	C28—C29—C30	125.1 (2)
C10—C11—C16	125.8 (2)	C29—C30—C31	110.0 (2)
S1—C12—C13	122.2 (2)	C30—C31—C32	112.7 (3)
C11—C12—C13	125.3 (2)	C31—C32—C33	113.0 (3)
S1—C12—C11	112.46 (17)	C28—C33—C32	110.2 (2)
C12—C13—C14	108.9 (2)	N4—C34—C27	177.3 (3)
C13—C14—C15	113.2 (3)	C18—C19—H19	122.00
C14—C15—C16	113.7 (3)	C20—C19—H19	122.00
C11—C16—C15	111.1 (2)	C21—C22—H22	122.00
N2—C17—C10	179.1 (3)	C23—C22—H22	122.00
C1—C2—H2	119.00	C18—C23—H23	119.00
C3—C2—H2	119.00	C22—C23—H23	119.00
C4—C3—H3	122.00	O3—C24—H24A	110.00
C2—C3—H3	122.00	O3—C24—H24B	110.00
C1—C6—H6	121.00	O4—C24—H24A	110.00
C5—C6—H6	121.00	O4—C24—H24B	110.00
O1—C7—H7B	110.00	H24A—C24—H24B	108.00
O2—C7—H7A	110.00	N3—C25—H25	118.00
O2—C7—H7B	110.00	C18—C25—H25	118.00
H7A—C7—H7B	108.00	C29—C30—H30A	110.00
O1—C7—H7A	110.00	C29—C30—H30B	110.00
N1—C8—H8	119.00	C31—C30—H30A	110.00
C1—C8—H8	119.00	C31—C30—H30B	110.00
H13A—C13—H13B	108.00	H30A—C30—H30B	108.00
C14—C13—H13A	110.00	C30—C31—H31A	109.00
C14—C13—H13B	110.00	C30—C31—H31B	109.00
C12—C13—H13A	110.00	C32—C31—H31A	109.00
C12—C13—H13B	110.00	C32—C31—H31B	109.00
H14A—C14—H14B	108.00	H31A—C31—H31B	108.00
C13—C14—H14A	109.00	C31—C32—H32A	109.00
C13—C14—H14B	109.00	C31—C32—H32B	109.00
C15—C14—H14A	109.00	C33—C32—H32A	109.00
C15—C14—H14B	109.00	C33—C32—H32B	109.00
C14—C15—H15B	109.00	H32A—C32—H32B	108.00
C16—C15—H15A	109.00	C28—C33—H33A	110.00
C14—C15—H15A	109.00	C28—C33—H33B	110.00
C16—C15—H15B	109.00	C32—C33—H33A	110.00
H15A—C15—H15B	108.00	C32—C33—H33B	110.00
H16A—C16—H16B	108.00	H33A—C33—H33B	108.00
			
C12—S1—C9—N1	−176.63 (19)	C9—C10—C11—C12	1.2 (3)
C12—S1—C9—C10	1.35 (17)	C17—C10—C11—C16	1.7 (3)
C9—S1—C12—C11	−0.70 (18)	C10—C11—C12—C13	−179.0 (2)
C9—S1—C12—C13	178.2 (2)	C16—C11—C12—S1	178.43 (17)
C26—S2—C29—C30	179.1 (2)	C10—C11—C12—S1	−0.1 (2)
C26—S2—C29—C28	0.38 (19)	C10—C11—C16—C15	167.3 (2)
C29—S2—C26—N3	178.9 (2)	C12—C11—C16—C15	−11.1 (3)
C29—S2—C26—C27	0.14 (18)	C16—C11—C12—C13	−0.4 (4)
C7—O1—C5—C6	−179.5 (2)	C11—C12—C13—C14	−15.8 (3)
C5—O1—C7—O2	−0.7 (2)	S1—C12—C13—C14	165.4 (2)
C7—O1—C5—C4	0.1 (3)	C12—C13—C14—C15	44.9 (4)
C7—O2—C4—C3	178.4 (2)	C13—C14—C15—C16	−60.2 (4)
C7—O2—C4—C5	−0.9 (3)	C14—C15—C16—C11	40.6 (4)
C4—O2—C7—O1	1.0 (2)	C23—C18—C19—C20	−0.3 (3)
C24—O3—C21—C22	179.8 (3)	C25—C18—C19—C20	178.9 (2)
C21—O3—C24—O4	0.0 (3)	C19—C18—C23—C22	0.8 (4)
C24—O3—C21—C20	0.2 (3)	C25—C18—C23—C22	−178.5 (2)
C20—O4—C24—O3	−0.2 (3)	C19—C18—C25—N3	0.6 (4)
C24—O4—C20—C21	0.3 (3)	C23—C18—C25—N3	179.8 (2)
C24—O4—C20—C19	−179.7 (3)	C18—C19—C20—O4	179.9 (2)
C8—N1—C9—C10	−171.7 (2)	C18—C19—C20—C21	−0.1 (4)
C8—N1—C9—S1	6.0 (3)	O4—C20—C21—O3	−0.4 (3)
C9—N1—C8—C1	179.13 (19)	O4—C20—C21—C22	−179.9 (2)
C25—N3—C26—C27	−178.1 (2)	C19—C20—C21—O3	179.7 (2)
C25—N3—C26—S2	3.4 (3)	C19—C20—C21—C22	0.1 (4)
C26—N3—C25—C18	−177.7 (2)	O3—C21—C22—C23	−179.2 (2)
C2—C1—C6—C5	0.3 (3)	C20—C21—C22—C23	0.4 (4)
C8—C1—C2—C3	−178.9 (2)	C21—C22—C23—C18	−0.8 (4)
C6—C1—C2—C3	0.6 (3)	S2—C26—C27—C28	−0.6 (3)
C8—C1—C6—C5	179.8 (2)	S2—C26—C27—C34	−179.41 (18)
C2—C1—C8—N1	171.9 (2)	N3—C26—C27—C28	−179.4 (2)
C6—C1—C8—N1	−7.6 (3)	N3—C26—C27—C34	1.8 (4)
C1—C2—C3—C4	−0.8 (4)	C26—C27—C28—C29	0.9 (3)
C2—C3—C4—C5	0.2 (4)	C26—C27—C28—C33	−179.0 (2)
C2—C3—C4—O2	−179.1 (2)	C34—C27—C28—C29	179.7 (2)
O2—C4—C5—O1	0.5 (3)	C34—C27—C28—C33	−0.2 (4)
O2—C4—C5—C6	−179.9 (2)	C27—C28—C29—S2	−0.8 (3)
C3—C4—C5—O1	−178.9 (2)	C27—C28—C29—C30	−179.5 (2)
C3—C4—C5—C6	0.7 (4)	C33—C28—C29—S2	179.15 (19)
C4—C5—C6—C1	−0.9 (3)	C33—C28—C29—C30	0.4 (4)
O1—C5—C6—C1	178.6 (2)	C27—C28—C33—C32	163.9 (2)
N1—C9—C10—C11	176.37 (18)	C29—C28—C33—C32	−16.0 (4)
N1—C9—C10—C17	−2.7 (3)	S2—C29—C30—C31	168.2 (2)
S1—C9—C10—C17	179.30 (17)	C28—C29—C30—C31	−13.2 (4)
S1—C9—C10—C11	−1.7 (2)	C29—C30—C31—C32	42.1 (3)
C9—C10—C11—C16	−177.3 (2)	C30—C31—C32—C33	−61.1 (4)
C17—C10—C11—C12	−179.8 (2)	C31—C32—C33—C28	45.3 (4)

### Hydrogen-bond geometry (Å, °)

**Table:**

*D*—H···*A*	*D*—H	H···*A*	*D*···*A*	*D*—H···*A*
C8—H8···S1	0.93	2.65	3.081 (2)	109
C25—H25···S2	0.93	2.61	3.060 (2)	110
C7—H7A···N4^i^	0.97	2.62	3.190 (3)	118

Symmetry codes: (i) *x*, *y*−1, *z*.
